# Meetings of Societies

**Published:** 1920-03

**Authors:** 


### MEETINGS OF SOCIETIES.

### Bristol Medico-Chirurgical Society.

*December 10th*, 1919.

I. Walker Hall, M.D., President, in the Chair.

Major F. P. Mackie, I.M.S., gave (by invitation) an address on **Disease in Mesopotamia.**

Dr. D. S. Davies opened a discussion on **The problem of malaria under post-war conditions.** The following took part in the discussion—Dr. S. J. Kerfoot, Dr. Cecil Clarke and Dr. Nierenstein (by invitation).

The following altered form of Rule 7, as passed at the last meeting, was confirmed :—

> "The Subscription to the Society shall be One and a Half Guineas per annum, payable in advance ; this sum shall include the subscription to the *Journal* and the use of the Library, with the privileges appertaining to the circulation of Library books."

---

*January 14th*, 1920.

I. Walker Hall, M.D., President, in the Chair.

Mr. A. J. Wright read notes on and showed two cases of **Congenital post-nasal stenosis.**

Mr. A. L. Flemming showed **A simple type of dropper for the administration of open ether.**

Dr. F. H. Edgeworth read notes on some cases of **Lethargic encephalitis.**

Dr. J. O. Symes read notes on a case of **Pancreatic insufficiency.**

---

*February 11th*, 1920.

I. Walker Hall, M.D., President, in the Chair.

Dr. G. A. Buckmaster gave an Address on **Additions to our knowledge of the physiology and pathology of the blood.**

*March* 10*th*, 1920.

I. WALKER HALL, M.D., President, in the Chair.

Dr. E. C. WILLIAMS showed (1) **A case of Muscular Dystrophy;** (2) **A case of hemorrhagic encephalitis with post-diphtheritic paralysis;** (3) **A case of diplegia.**

Dr. CAREY COOMBS read notes on **Irritable heart in civil practice.**

Dr. C. E. K. HERAPATH read notes on **Irritable heart in pensioners.**

Dr. A. G. MORRIS (by invitation) read notes on **Irritable heart in soldiers.**

Dr. A. BIRRELL (by invitation) read notes on **Post-influenzal tachycardia.**

